# Humoral immunity to SARS-CoV-2 and seasonal coronaviruses in children and adults in north-eastern France

**DOI:** 10.1016/j.ebiom.2021.103495

**Published:** 2021-07-23

**Authors:** Tom Woudenberg, Stéphane Pelleau, François Anna, Mikael Attia, Françoise Donnadieu, Alain Gravet, Caroline Lohmann, Hélène Seraphin, Raphaël Guiheneuf, Catherine Delamare, Karl Stefic, Julien Marlet, Etienne Brochot, Sandrine Castelain, Olivier Augereau, Jean Sibilia, François Dubos, Damia Meddour, Christèle Gras-Le Guen, Marianne Coste-Burel, Berthe-Marie Imbert-Marcille, Anne Chauvire-Drouard, Cyril Schweitzer, Amélie Gatin, Sandra Lomazzi, Aline Joulié, Hervé HAAS, Aymeric Cantais, Frederique Bertholon, Marie-France Chinazzo-Vigouroux, Mohamed SI Abdallah, Laurence Arowas, Pierre Charneau, Bruno Hoen, Caroline Demeret, Sylvie Van Der Werf, Arnaud Fontanet, Michael White

**Affiliations:** a Infectious Disease Epidemiology and Analytics Unit, Department of Global Health, Institut Pasteur, Paris, France; b Malaria: Parasites and Hosts Unit, Department of Parasites and Insect Vectors, Institut Pasteur, Paris, France; c Molecular Virology and Vaccinoloy Unit, Department of Virology, Institut Pasteur, Paris, France; d Molecular Genetics of RNA Viruses, Department of Virology, Institut Pasteur, CNRS UMR 3569, Paris, France; e Laboratoire de Microbiologie, Groupement Hospitalier Régional de Mulhouse et Sud-Alsace, Mulhouse, France; f Centre Hospitalier Simone Veil de Beauvais, Beauvais, France; g CHR Metz Thionville, Metz, France; h Service de Bactériologie-Virologie, Hôpital Bretonneau, CHRU de Tours, Tours, France; i Service de Virologie, CHU Amiens Picardie, UR 4294 AGIR UPJV, Amiens, France; j Service de Microbiologie, Hôpitaux Civils de Colmar, Colmar, France; k Laboratoire de Virologie, CHU de Strasbourg, Strasbourg, France; l Univ. Lille, CHU Lille, Urgences pédiatriques et maladies infectieuses, Lille, France; m Urgences Pédiatrique et Pédiatrie Générale Hopital Mère Enfant CHU de Nantes, Nantes, France; n Service de Virologie CHU Nantes, Nantes, France; o CHU Nantes, CIC FEA1413, INSERM, Nantes, France; p Hôpital d'Enfants, CHRU de Nancy, Vandoeuvre-Les-Nancy, France; q EA 3450, DevAH, Université de Lorraine, Vandoeuvre Lès Nancy, France; r Pediatric Emergency Unit, Hôpital d'enfants, CHRU Nancy; s CRBL, CHRU Nancy, Nancy, France; t Urgences pédiatriques et pédiatrie générale, hôpitaux pédiatriques CHU Lenval, Nice; u Pediatric Emergency Department, Hospital University of St Etienne, France; v Urgences pédiatriques Hopital Clocheville, CHRU de Tours, Tours, France; w Agence régionale de santé Hauts-de-France; x Investigation Clinique et Accès aux Ressources Biologiques (ICAReB), Center for Translational Research, Institut Pasteur, Paris, France; y Direction de la recherche médicale, Institut Pasteur, Paris, France; z National Reference Center for Respiratory Viruses, Institut Pasteur, Paris, France; aa Emerging Diseases Epidemiology Unit, Department of Global Health, Institut Pasteur, Paris, France; ab PACRI Unit, Conservatoire National des Arts et Métiers, Paris, France

**Keywords:** SARS-CoV-2, COVID-19, seroprevalence, sero-epidemiology, seasonal coronaviruses, antibody response

## Abstract

**Background:**

Children are underrepresented in the COVID-19 pandemic and often experience milder disease than adolescents and adults. Reduced severity is possibly due to recent and more frequent seasonal human coronaviruses (HCoV) infections. We assessed the seroprevalence of SARS-CoV-2 and seasonal HCoV specific antibodies in a large cohort in north-eastern France.

**Methods:**

In this cross-sectional seroprevalence study, serum samples were collected from children and adults requiring hospital admission for non-COVID-19 between February and August 2020. Antibody responses to SARS-CoV-2 and seasonal HCoV (229E, HKU1, NL63, OC43) were assessed using a bead-based multiplex assay, Luciferase-Linked ImmunoSorbent Assay, and a pseudotype neutralisation assay.

**Findings:**

In 2,408 individuals, seroprevalence of SARS-CoV-2-specific antibodies was 7-8% with three different immunoassays. Antibody levels to seasonal HCoV increased substantially up to the age of 10. Antibody responses in SARS-CoV-2 seropositive individuals were lowest in adults 18-30 years. In SARS-CoV-2 seronegative individuals, we observed cross-reactivity between antibodies to the four HCoV and SARS-CoV-2 Spike. In contrast to other antibodies to SARS-CoV-2, specific antibodies to sub-unit 2 of Spike (S2) in seronegative samples were highest in children. Upon infection with SARS-CoV-2, antibody levels to Spike of betacoronavirus OC43 increased across the whole age spectrum. No SARS-CoV-2 seropositive individuals with low levels of antibodies to seasonal HCoV were observed.

**Interpretation:**

Our findings underline significant cross-reactivity between antibodies to SARS-CoV-2 and seasonal HCoV, but provide no significant evidence for cross-protective immunity to SARS-CoV-2 infection due to a recent seasonal HCoV infection. In particular, across all age groups we did not observe SARS-CoV-2 infected individuals with low levels of antibodies to seasonal HCoV.

**Funding:**

This work was supported by the « URGENCE COVID-19 » fundraising campaign of Institut Pasteur, by the French Government's Investissement d'Avenir program, Laboratoire d'Excellence Integrative Biology of Emerging Infectious Diseases (Grant No. ANR-10-LABX-62-IBEID), and by the REACTing (Research & Action Emerging Infectious Diseases), and by the RECOVER project funded by the European Union's Horizon 2020 research and innovation programme under grant agreement No. 101003589, and by a grant from LabEx IBEID (ANR-10-LABX-62-IBEID).

**Research in context**

**Evidence before this study**

We searched PubMed on April 15, 2021 using the terms (“SARS-CoV-2” OR “COVID-19”) AND (“antibody” OR “humoral immunity”) AND (“seasonal coronavirus” OR “OC43” OR “NL63” OR “HKU1” OR “229E”) to assess cross-reactivity between antibody responses to SARS-CoV-2 and seasonal coronaviruses. Our search showed 57 publications. Cross-reactivity in pre-pandemic samples between SARS-CoV-2 and seasonal coronaviruses is generally low and antigen-dependent. Higher degree of cross-reactivity was observed with subunit S2 of Spike, which was found to be neutralising in one study. A few studies reported enhanced anti-Spike antibody levels to OC43 and/or HKU1 in SARS-CoV-2 infected individuals. However, most of the studies looked at antibody responses with a relatively small sample size or limited the measurement to adults.

**Added value of this study**

Our study provides a detailed characterization of humoral immunity to SARS-CoV-2 and seasonal coronaviruses in a study population comprising a wide age spectrum including one of the largest collection of samples from children assembled to date. We measured antibodies to different antigens with two assays and a pseudotype neutralisation assay. We found that the immune response to SARS-CoV-2 is lower in adolescents and young adults compared to young children and older adults. We found clear evidence for serological cross-reactivity, most notably for the betacoronavirus OC43. We found that SARS-CoV-2 infection induces higher anti-OC43 Spike IgG responses, and conversely that OC43 infection likely induces higher anti-SARS-CoV-2 IgG responses.

**Implications of all the available evidence**

A previous seasonal coronavirus infection seems limited to cross-reactivity in assays rather than cross-protection from a SARS-CoV-2 infection. An elevated antibody response to spike subunit 2 in young children is remarkable. For a definitive answer whether an infection with seasonal coronavirus induces cross-protection, longitudinal studies are necessary.

Alt-text: Unlabelled box

## Introduction

Severe acute respiratory syndrome coronavirus 2 (SARS-CoV-2) has infected at least 100 million people since its discovery [1] and, as of February 2021, has led to almost 2.5 million deaths [2]. SARS-CoV-2 belongs to the *Coronaviridae* family in which 7 coronaviruses are known to exist associated with respiratory tract infections in humans [3]. Four of these are endemic to humans, including 2 alphacoronaviruses, 229E and NL63, and two betacoronaviruses OC43 and HKU1. SARS-CoV-2, as well as SARS-CoV and Middle East respiratory syndrome coronavirus (MERS-CoV), are betacoronaviruses.

Whereas the epidemiology of SARS-CoV and MERS-CoV is characterized by small and contained epidemics, seasonal human coronaviruses (HCoV) are endemic around the world [4]. After a small decrease in the seroprevalence to all four seasonal HCoV due to waning of maternal antibodies [5], seroprevalence rises rapidly in childhood, after which it remains stable in adults [[6], [7], [8]]. First infection with seasonal coronaviruses typically occurs within 5 years of birth [5,8]. Regular re-exposures to seasonal HCoV occur throughout life [9].

While the vast majority of children experience their first seasonal HCoV infection at an early age, children are underrepresented among the number of reported cases of coronavirus disease 2019 (COVID-19) [10,11]. The underrepresentation of children in the number of COVID-19 cases can be explained by a reduced susceptibility to infection as well as a reduced probability of developing symptoms and severe COVID-19 among children compared with adults [[11], [12], [13], [14], [15]]. The percentage of symptomatic cases is estimated to be around 20% for 10 to 19 year olds [13], whereas the percentage of infections showing clinical manifestations rises to 69% in people over 70. The reduced disease susceptibility and severity remains poorly understood, but may be due to cross-protection derived from previous seasonal HCoV infections [[16], [17], [18]].

There are limited cross-reactive antibody responses against SARS-CoV-2 in pre-pandemic samples [[19], [20], [21]], with cross-reactive antibodies targeting Nucleocapsid protein occasionally reported [20,22]. Cross-reactivity in pre-pandemic samples has not been found to be associated with neutralising activity [20,23]. Cross-reactivity in samples from adults with a recent endemic HCoV infection was also limited [19,24]. Yet, when Ng *et al*. examined pre-pandemic samples from children, 21 out of 48 showed IgG antibody responses (either targeted to Spike or Nucleocapsid) that harbored moderate SARS-CoV-2 neutralising activity [24]. Whether this neutralising activity is due to previous HCoV infections is still unclear, with several studies finding little difference between SARS-CoV-2 seropositive and seronegative children in HCoV antibodies [15,25].

In this study, comprising a large number of serum samples from children and adults hospitalized for other reasons than COVID-19 , we aimed to measure exposure to seasonal coronaviruses and to SARS-CoV-2 using three different immunoassays, a bead-based multiplex serological [26] assay, a Luciferase-Linked ImmunoSorbent Assay (LuLISA, [27,28] and a pseudo neutralisation assay [27,28].

In this detailed analysis of humoral immunity in a large cohort of French children, we demonstrate that children of all ages are susceptible to infection with SARS-CoV-2, and have comparable antibody responses to adults. The lowest antibody levels were observed in adults aged 18-30 years. In this cross-sectional study, there was no significant evidence for prior exposure to seasonal coronaviruses being associated with protection against SARS-CoV-2 infection.

## Methods

### Study design

In this cross-sectional study, we aimed to measure the seroprevalence of antibodies targeting SARS-CoV-2 and seasonal HCoV among children attending hospitals in north-eastern France and adults in two hospitals. Serum samples were collected from February 2020 to August 2020 and were analysed with three different immunoassays. A subset of the samples (n = 90) were collected prior to the COVID-19 pandemic between 2002 and 2019.

### Study population

Study population is composed of individuals with an age ranging from 0 to 100 years. Analysed samples were either anonymous residual serum samples from medical care or samples collected in other clinical studies (INCOVPED NCT04336761) after informed consent. Information on age, sex and date of sampling were collected from medical records or study databases and compiled with the serological results. The use of those samples complies with the applicable ethical principles and regulatory requirements including the GDPR ones.

### Ethics Statement

The majority of the serum samples analysed in this study (2404/2544) were leftovers from routine medical blood sample processing in French hospital laboratories. They were processed in accordance with existing regulations and guidelines of the French Commission for Data Protection (Commission Nationale de l'Informatique et des Libertés). Sera were completely anonymous, and it was not possible toreturn to individual patients' files. According to the French law, no informed consent is required for processing such samples.

Other samples (141/2544) were collected for the purpose of a clinical study (INCOVPED, which is registered with ClinicalTrials.gov [NCT04336761]). When signing the informed consent form for this study, participants' parents had been informed that and had consented to collected samples could be used for other approved research studies

### Serological assays

All serum samples were tested for IgG antibody responses to SARS-CoV-2 using three assays. First, a bead-based 9-plex assay (Luminex) tested for IgG antibodies to five SARS-CoV-2 antigens (trimeric Spike ectodomain, Receptor Binding Domain (RBD), Spike S2 subunit, Nucleocapsid, and Membrane–Envelope fusion) and against trimeric Spike of the four different seasonal coronaviruses (229E, HKU1, NL63, OC43). Second, the Luciferase-Linked ImmunoSorbent assay (LuLISA) detected antibodies directed against SARS-CoV-2 Nucleocapsid. Third, a pseudoneutralisation (pseudo NT) assay was used to detect SARS-CoV-2 neutralising antibodies.

### Bead-based multiplex assay

In a 96 well, non-binding microtiter plate 50 uL of protein-conjugated magnetic beads (500/region/uL) and 50 uL of diluted serum were mixed using a pipette and incubated for 30 min at room temperature on a plate shaker. All dilutions were made in phosphate buffered saline containing 1% bovine serum albumin and 0.05% (v/v) Tween-20 (denoted as PBT), and all samples were run in singlicate. Following incubation, the magnetic beads were separated using magnetic plate separator (Luminex®) for 60 seconds and washed thrice with 100 μl PBT using a multichannel pipette. The washed magnetic beads were incubated for 15 minutes with detector secondary antibody at room temperature on a plate shaker. The magnetic beads were separated and washed thrice with 100 μl PBT and finally resuspended in 100 μL of PBT. Samples were diluted at 1/100, and R-Phycoerythrin-(R-PE) conjugated Donkey Anti-Human IgG antibody was used as detector antibody at 1/120 dilution. A positive control pool of serum at two-fold serial dilutions from 1:50 to 1:102,400 was included on each 96 well plate. Plates were read using a Luminex® MAGPIX® system, which provides a reading of median fluorescence intensity (MFI). A 5-parameter logistic curve was used to convert measurements from MFI to relative antibody units (RAU). RAU were used in a random forests algorithm to determine seropositivity.

### Luciferase-Linked ImmunoSorbent Assay

Briefly, Nucleocapsid-specific IgG antibodies were assessed using an ELISA‐based assay on sera incubated in antigen‐coated wells. Antigens have been produced as follows. Full‐length N protein from SARS‐CoV‐2 were produced with a (His)_6_ tag in the *E. coli*, purified on Ni‐NTA affinity column, and then size‐exclusion chromatography was performed. White 384‐well plates with flat bottoms (Fluoronunc C384 Maxisorp, Nunc) were coated with 1 μg/mL of Nucleocapsid protein in PBS buffer, 50 μL/well for 3 h at room temperature, or overnight at 4°C. Wells were washed using a plate washer (Zoom, Berthold Technologies, Germany) two cycles of three times with 100 μL of PBS/Tween 20 0.1%. Sera were diluted 200 times in PBS, nonfat milk 1%, and Tween 20 0.1%. 50 μL of serum dilutions were incubated for 1 h at room temperature in their respective wells. Wells were washed two cycles of three times with 100 μL of PBS/Tween 20 0.1%. The Anti‐Fc IgG VHH (Fc1) was derived from an antibody from immunized alpaca and expressed as a tandem with an optimized catalytic domain nanoKAZ from *Oplophorous gracilirostris* luciferase. Purified Fc1‐nanoKAZ 1 ng/mL (400 × 10^6^ RLU·s^–1^·mL^–1^) in PBS, nonfat milk 1%, and Tween 20 0.1% was loaded (50 μL/well) and incubated for 30 min at room temperature. Wells were washed two cycles of three times with 100 μL of PBS/Tween 20 0.1% then 50 μL of the luciferin solution was added (Promega). Photons production was counted during 0.5 s per well and measured two times in a plate luminometer (Mithras2; Berthold, Wildbad, Germany). Positivity for LuLISA assay is defined by a cut-off eliminating at least 98% of pre-pandemic samples (10,291 RLU/s).

### Pseudotype neutralisation assay

SARS-CoV-2 S-Pseudotyped viruses were produced by transfection of 293T cells as previously described [29]. The pseudo neutralisation assay used in this study and its calibration are described in details elsewhere [27,28]. As pseudotyped particles express the full length Spike, this assay measures neutralization due to disruption of Spike/hACE2 interaction, therefore neutralization relies on antibodies targeting either S1 or S2 subdomains. Briefly, sera were decomplemented at 56°C during 30 min in a water bath and diluted at 1/40 then co‐incubated with 300 Transduction Units of a SARS-CoV-2 S pseudo‐typed vector at room temperature during 30 min under agitation. Mix is then plated in tissue culture treated black 96‐well plate clear bottom (Costar) with a suspension of 20 000 HEK 293T-hACE2 cells in culture medium DMEM‐glutamax (Gibco) + 10% FCS (Gibco) + Pen/Strep (Gibco). After 48‐h incubation at 37°C 5% CO2, bioluminescence is measured using a Luciferase Assay System (Promega) on an EnSpire plate reader (PerkinElmer). The mean of S pseudotypes -driven reporter activity (GFP or Luciferase) obtained after incubation of the pseudo types with negative pre-epidemic sera minus 3 standard deviation (mean prepdm -3SD) defines the threshold for positivity in pseudo NT. For each exploratory serum, a pseudoNT percentage is calculated by dividing the S pseudotypes reporter value by the threshold, to give the percentage of pseudoNT. A cut off of 6% PNT is set up as cut off for positivity in pseudoNT

### Statistical methods

A random forests algorithm was developed to determine seropositivity based on relative antibody units measured with the 9-plex assay. This algorithm was trained on samples from both PCR-confirmed cases of COVID-19 and negative control samples, and calibrated to have 99% specificity [26]. Further details on the development of this algorithm can be found in the appendix. The cut-off between seropositive and seronegative samples with the pseudoneutralisation assay and the LuLISA assay was determined using large panels of negative controls to obtain 99% specificity. Samples were measured in duplicate with the LuLISA assay, and corresponded very well with a Pearson's correlation estimate of 0.96 (Supplement Figure 1).

**Fig. 1:**
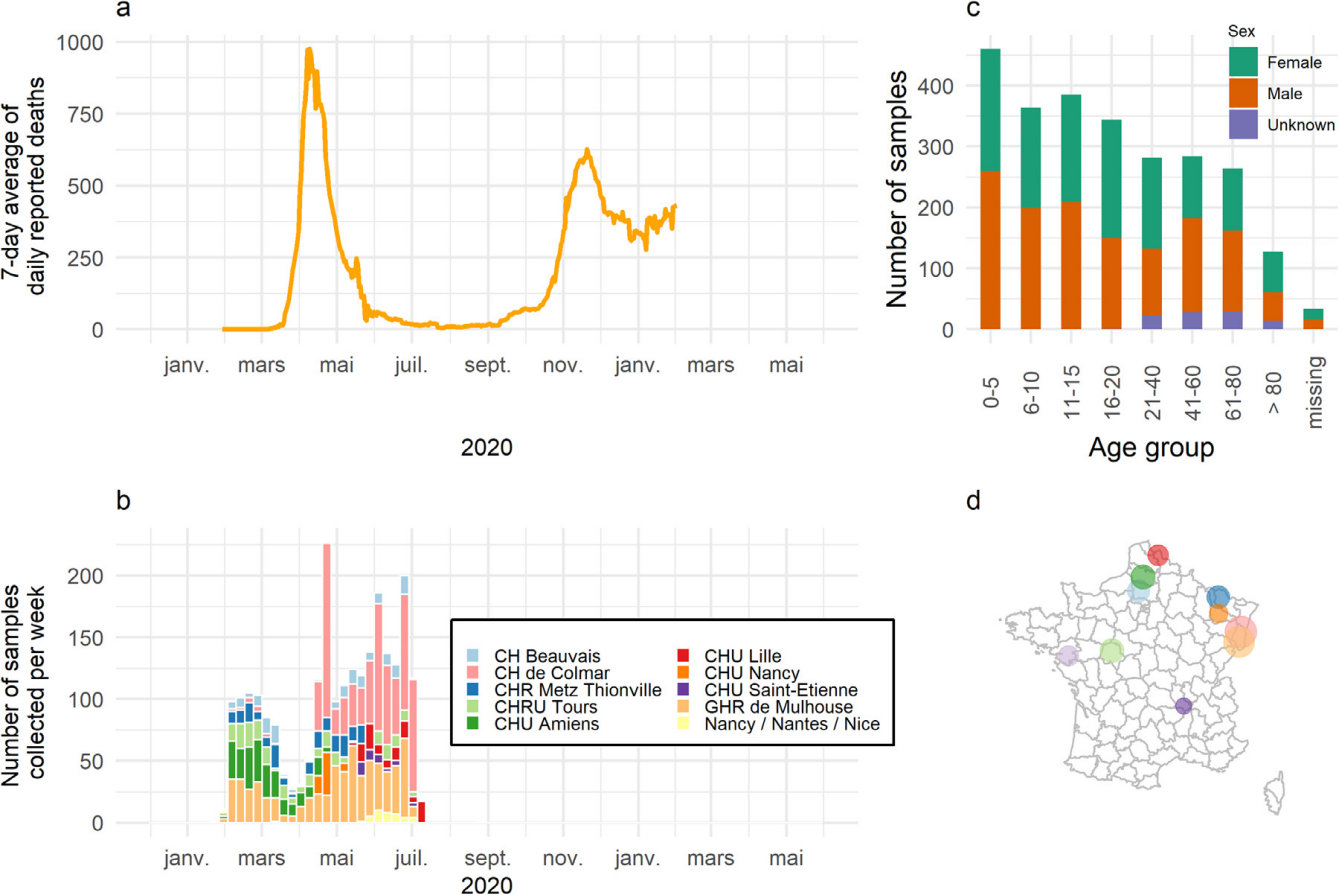
Epidemiological context and description of sampling (n = 2544). (a, b) Collection of samples occurred during the first wave of COVID-19 in France. (c) Majority of sera were drawn from children below 20. (b,d) Samples were collected from 11 hospitals located predominantly in north-eastern France.

The performance of and concordance between the three assays was investigated by assessing the overall, positive, and negative agreement, and Cohen's Kappa statistic. In addition, we re-assessed the specificity of our three assays with 90 samples, which were collected prior to 2020.

SARS-CoV-2 antibody prevalence was analysed using descriptive statistics: number and proportions for discrete variables, and median and ranges for continuous variables. Seroprevalence rates between age groups, sites, and sex were compared using the Chi-squared test. Binomial confidence intervals around the seroprevalence estimates were estimated using Wilson's method. We used the Locally Weighted Scatterplot Smoothing (LOWESS) method to visualize the relationship between relative antibody units and age. It is a non-parametric method where least squares regression is performed in local subsets. We used R version 4.0.3 to conduct all analyses.

### Role of the funding source

The funders had no role in study design, data collection, data analyses, interpretation, or writing of the report.

## Results

In total, 2544 participants were recruited. Sera were sampled throughout the first wave of COVID-19 in France (Figure 1a). Samples originated from 11 hospitals located predominantly in North and East France (Table 1). Two hospitals included adults in addition to children in the recruitment (CHU Colmar and CHU Mulhouse). 40 samples collected from children in hospitals in Nice, Nantes and Nancy as part of a study entitled INCOVPED were grouped for analysis. Most participants were below 19 years of age (n = 1505, 60%) and 52% were male.

**Table 1:** Demographic characteristics of 2544 subjects sampled predominantly in north-eastern France, 2020

	N	%

Sex		
Female	1173	48
Male	1276	52
Missing	95	
Date of sampling		
Pre-pandemic	90	4
February 2020 and onwards	2449	96
Missing	5	
Age		
0-5	460	18
6-10	364	14
11-15	385	15
16-20	344	14
21-40	282	11
41-60	284	11
61-80	264	11
>80	127	5
Missing	34	
Hospital		
CH Beauvais	159	7
CHU Amiens	237	9
CH de Colmar	767	30
GHR de Mulhouse	676	27
CHU Nancy	57	2
CHU Nantes	91	4
CHRU Tours	204	8
CHR Metz Thionville	175	7
CHU Lille	101	4
CHU Saint-Etienne	37	1
Nice / Nantes / Nancy (INCOVPED)	40	2

**Fig. 2:**
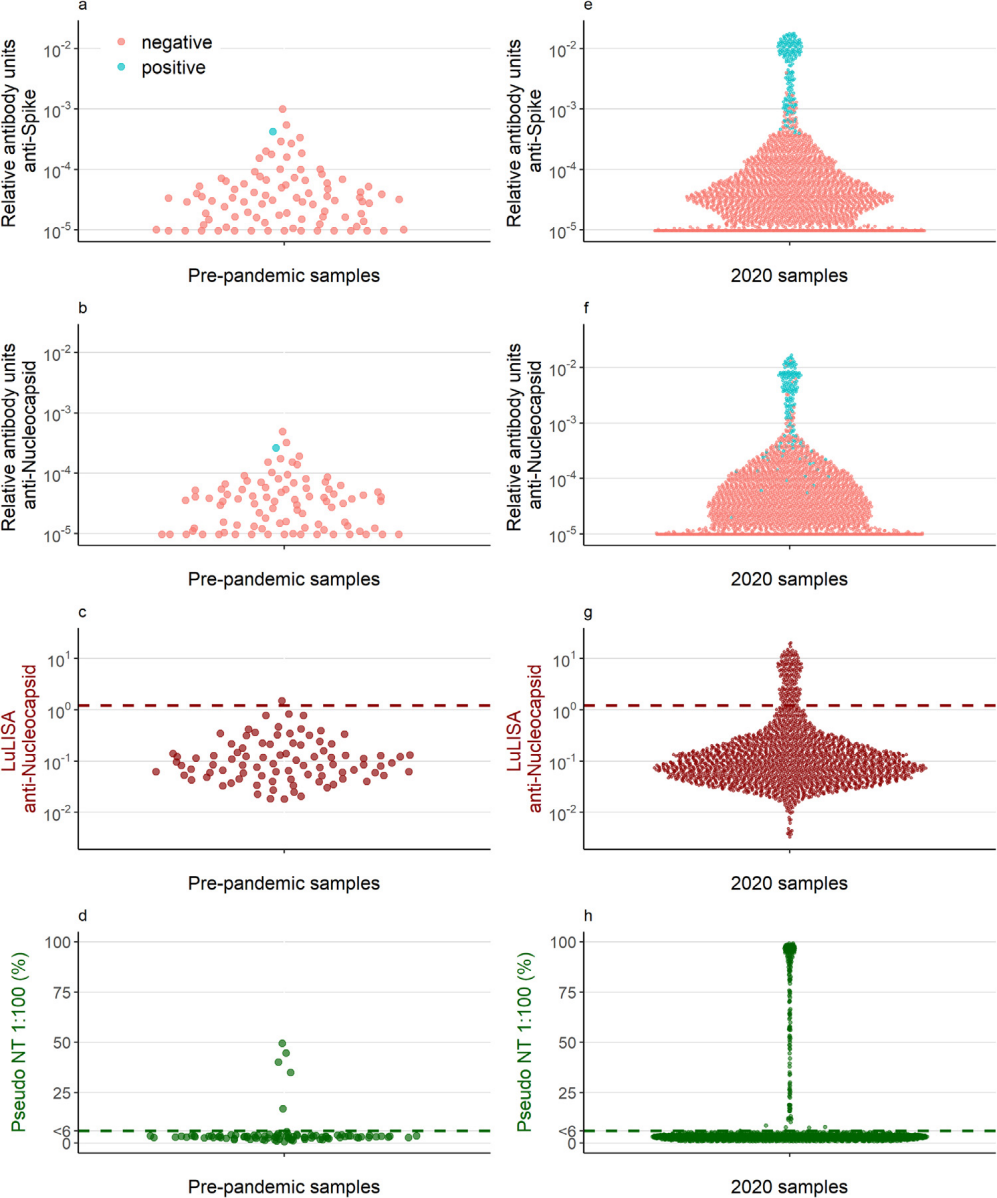
Antibody concentrations and neutralising activity of (a-d) pre-pandemic samples (n = 90) and (e-h) samples collected in 2020 (n = 2449). The dark red dashed line in panels c and g is the cut-off defining seropositivity for the Luciferase-Linked ImmunoSorbent Assay. The dark green line in panels d and h depicts the cut-off at 6%, which is the threshold for seropositivity in the pseudoneutralising assay. Samples below 6% were assigned the value of 3. The points in red (negative) and blue (positive) in panels a, b, e, and f depict seropositivity based on the random forest algorithm using antibody levels to Nucleocapsid, Spike, and Recepter Binding Domain measured by the Luminex bead-based multiplex assay.

As a subset of the pediatric samples were collected prior to 2020, we validated the specificity of our three assays. 1/90 pre-pandemic samples were classified positive with the 9-plex assay. 1/90 pre-pandemic samples tested positive for Nucleocapsid-specific antibodies on the LuLISA assay and 5/90 samples had neutralising activity of at least 6% (Figure 2a-d).

**Fig. 3:**
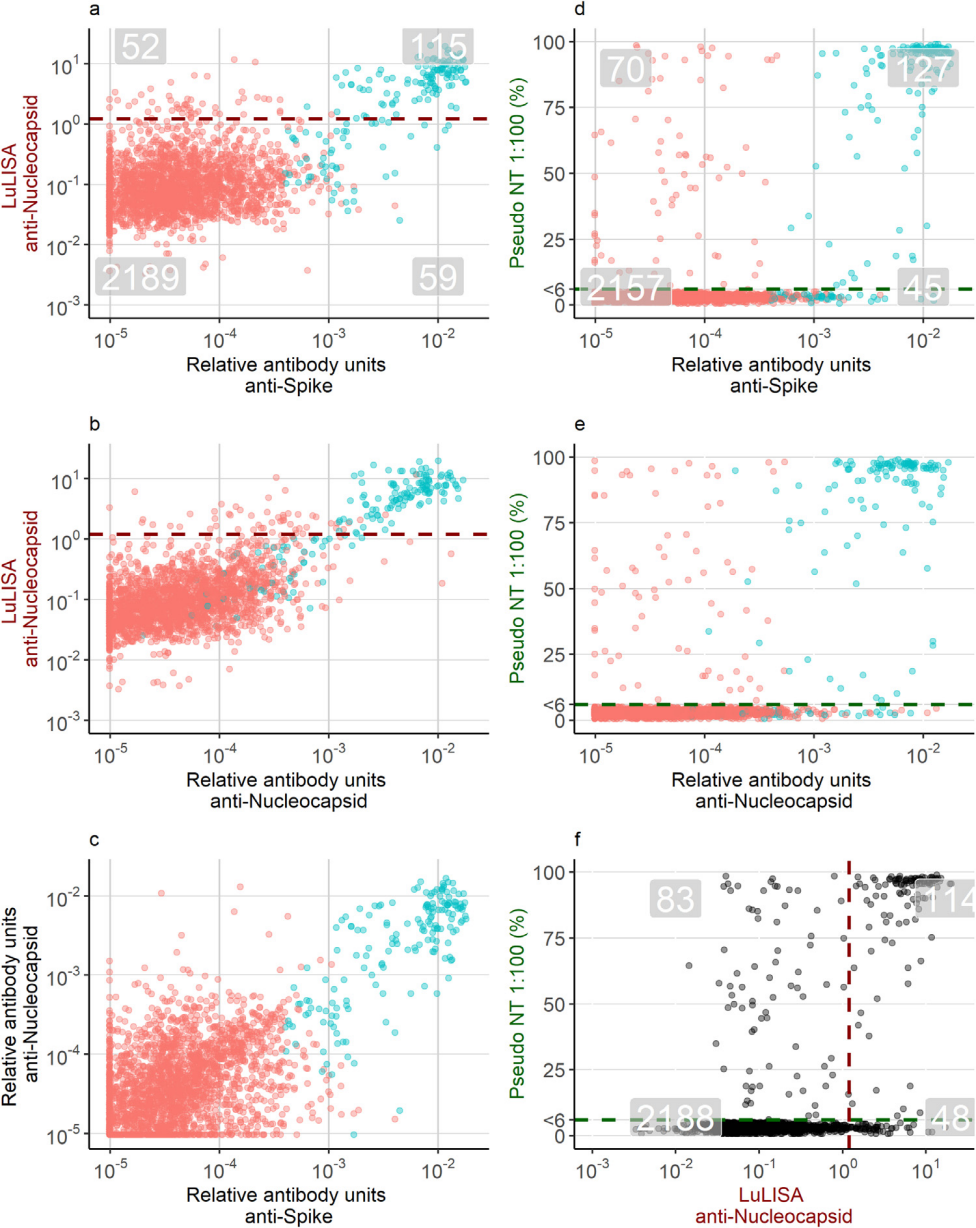
Pairwise comparison of 2449 serum samples in three immunoassays. Grey highlighted numbers in panels a, d, and f provide an assessment of the agreement between the immunoassays. Numbers located at the top-right are samples classified as positive in both assays, and numbers bottom-left are samples classified as negative in both assays. Numbers in the top-left or bottom-right are discordant classified samples between two tests. The dark red dashed line in panels a, b and f is the cut-off defining seropositivity for the Luciferase-Linked ImmunoSorbent Assay. The dark green line in panels d, e, and f depicts the cut-off at 6%, which is the threshold for seropositivity based on the pseudoneutralising assay. Samples below 6% were assigned the value of 3. The points in red (negative) and blue (positive) depict seropositivity based on the bead-based multiplex assay.

Antibody levels of samples collected since February 2020 are shown in Figure 2e-h. 7% (174/2415) samples had antibody levels measured with the 9-plex Luminex assay that were classified as positive using the random forests algorithm. 7% (167/2449) samples were classified as positive with the LuLISA assay. 8% (197/2433) samples had neutralising activity. The high specificity and the high number of negative samples led to a very high agreement between the different tests (Figure 3). 95% ((2188+114)/2433) samples were similarly classified between the LuLISA and the pseudoneutralisation assay, 95% ((2157 + 127)/2399) were similarly classified between the 9-plex and the pseudoneutralisation assay, and 95% ((2189 + 115)/2415) were similarly classified between the Luminex and the LuLISA. Notable were the samples, which had neutralising activity but were classified negative with the other two assays, suggesting some neutralising activity was unexplained by measured antigen-specific antibodies. A further assessment of the agreement between the three assays is provided as supplementary material (Supplement Table 1).

**Fig. 4:**
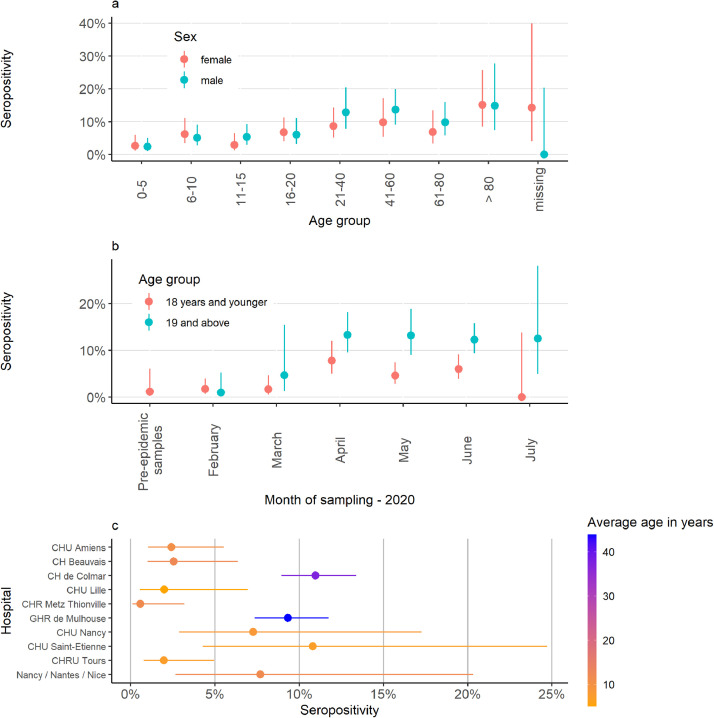
Epidemiological description of 2449 samples collected in North-eastern France, 2020. Panel a depicts the seroprevalence by age group and sex. In Panel b, the seroprevalence is stratified by month of sampling and in panel c by hospital.

Out of 2415 samples, 174 were classified as positive based on the random forests classification using Luminex measured antibody responses to three SARS-CoV-2 antigens (Spike, RBD, NP). This indicates an overall seroprevalence of 7.2%. Overall, seroprevalence was higher in men (7.7%) than in women (6.6%) (Figure 4a). Seroprevalence varied among the different hospitals, largely due to different sampling periods and recruited age groups (Figure 4b-c). Among samples collected from May onwards (111 out 1306, 8.5%) and among samples taken from adults (112 out of 1004, 11%) a higher proportion tested positive than samples collected before May (63 out of 1109, 5.7%) or taken from children (60 out of 1382, 4.3%). Samples from Mulhouse and Colmar had high seropositivity rates, possibly associated with the large share of the samples originating from adults or a consequence of the high levels of COVID-19 transmission in Alsace in Spring 2020 (Figure 4c). The seropositivity in samples from Saint-Etienne and Nancy was higher than average, consistent with a later timing of sampling compared with most other hospitals in our study (Figure 1b).

**Fig. 5:**
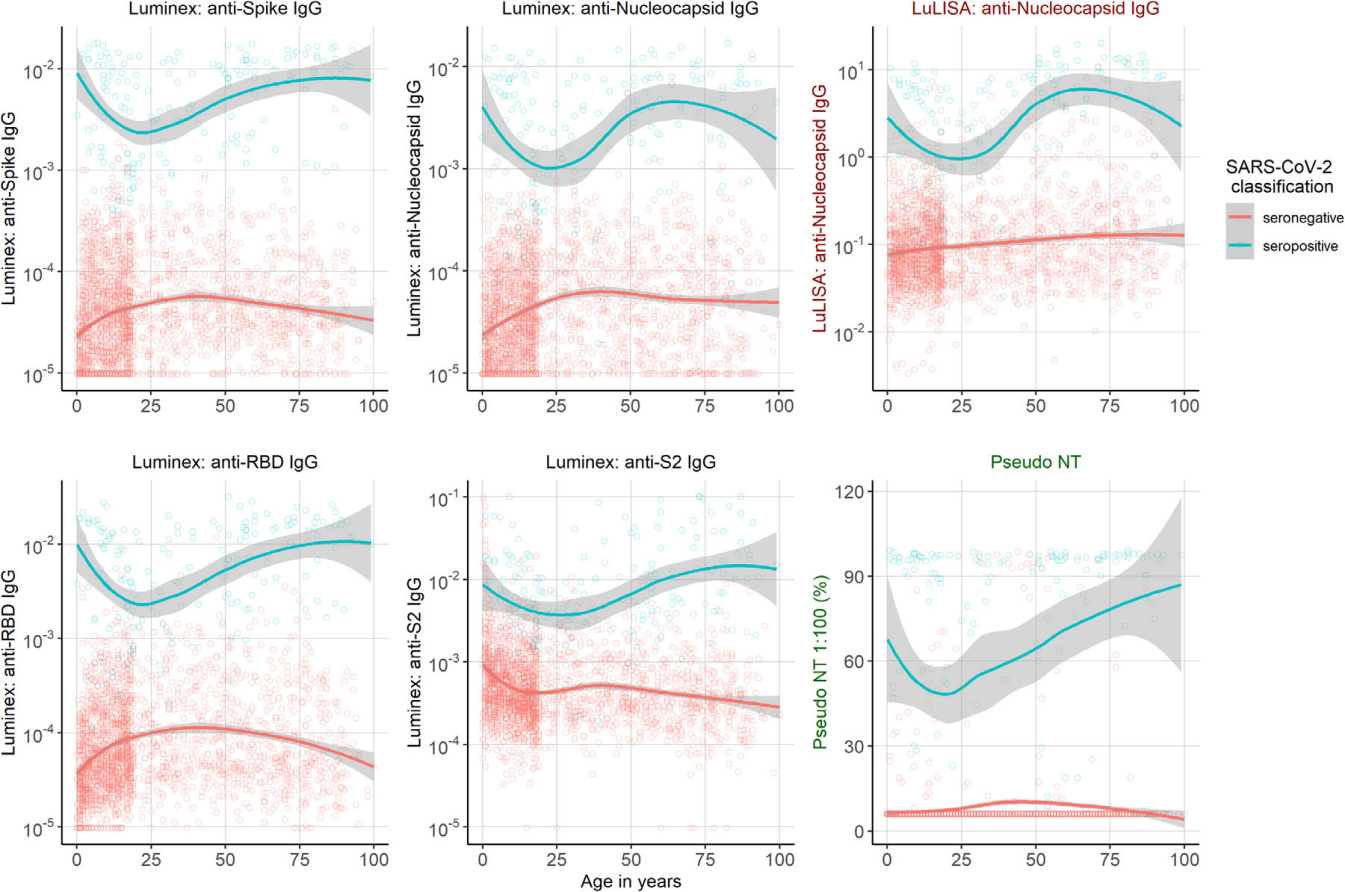
Antibody response to SARS-CoV-2 of 2449 samples collected in North-eastern France, 2020. Relative antibody units to four antigens shown by age measured with the LuLISA and the multiplex assay (Luminex). A random forests algorithm determined SARS-CoV-2 classification (positive in blue and negative in red). The smoothed line was fitted using the LOWESS method, with 95% confidence intervals denoted by the shaded region. The sixth graph shows the pseudo neutralisation activity by age.

The distribution of antibody responses by age and classification are shown in Figure 5. For positively classified samples (blue in Figure 5), antibody responses are highest in adults over 50 and lowest in young adults around the age of 25. Samples classified seronegative show an upward trend in the antibody responses going from infancy to adolescence, which may reflect cross-reactivity between antibodies to seasonal HCoV antibodies and SARS-CoV-2. Indeed there is a clear correlation between antibodies to Spike of the seasonal HCoV and SARS-CoV-2 Spike in seronegative individuals (Supplement Figure 2). For antibodies targeting sub-unit 2 of Spike, we observed an opposite trend with higher levels in seronegative children below 10 vs older age groups.

**Fig. 6:**
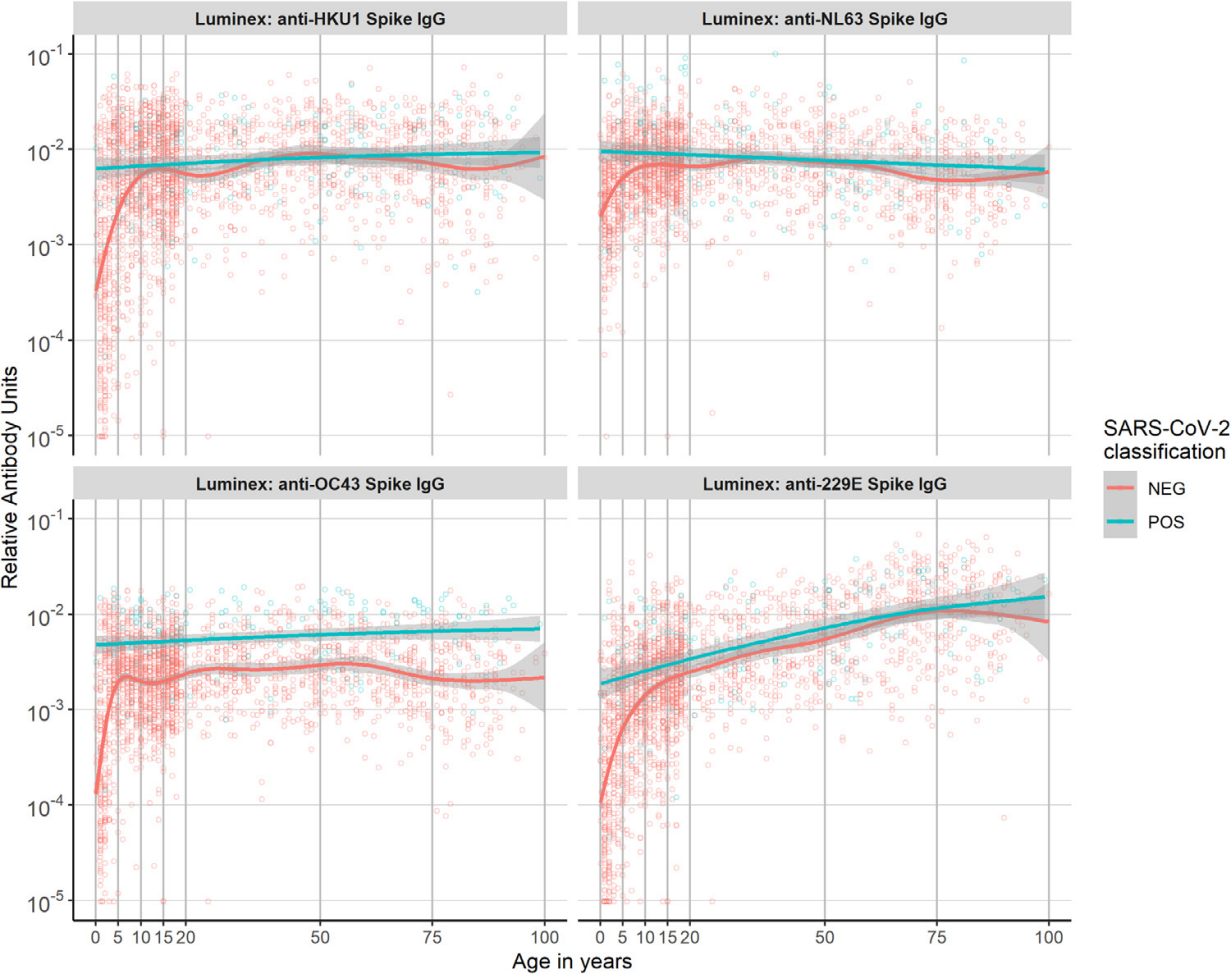
Relative antibody units to the four seasonal coronaviruses by age and seropositivity classification of 2449 samples collected in North-eastern France, 2020. The smoothed line was fitted using the LOWESS method, with 95% confidence intervals denoted by the shaded region.

In addition to antibodies to SARS-CoV-2, we also measured the antibody response to Spike of four seasonal coronaviruses (NL63, HKU1, OC43, and 229E). Antibodies to all seasonal coronaviruses increased substantially with age, and across the whole age range for 229E (Figure 6). Upon infection with SARS-CoV-2, antibody levels to Spike of betacoronavirus OC43 (but not other HCoV) increased across the whole age spectrum, which may reflect a higher affinity of SARS CoV2 antibodies for OC43 Spike antigen unobserved among the other seasonal HCoV. We observed no samples from individuals infected with SARS-CoV-2 with low levels of antibodies to seasonal coronaviruses. Below the age of five, samples classified as seropositive to SARS-CoV-2 had elevated levels of antibodies to all seasonal coronaviruses compared with seronegative samples. Finally, in seronegative individuals, there is a clear correlation between antibodies to the four HCoV and SARS-CoV-2 Spike (Supplement Figure 2).

## Discussion

In this study, we assessed the antibody responses to a variety of SARS-CoV-2 antigens and seasonal coronaviruses using three different immunoassays in a large cohort of French children and adults. SARS-CoV-2 infections occurred across the full age spectrum with higher seropositivity rates in adults and adolescents compared with children. Higher seroprevalence estimates were also observed in heavily affected areas during the first wave in France [30], and samples collected further in the year, reflecting the progression of the epidemic [31]. Within positive classified samples, the immune response to several antigens is lower in adolescents and young adults compared to young children and older adults. These age-related immune responses have been observed previously [32].

As expected [5], seroprevalence of antibodies to the four HCoV increased with age, most profoundly below the age of 10, and throughout life for 229E. We observed clear signals of cross-reactivity between SARS-CoV-2 and HCoV anti-Spike antibodies. Firstly, we found correlations between anti-Spike SARS-CoV-2 antibodies and anti-Spike HCoV antibodies in seronegative individuals providing evidence for cross-reactivity. Secondly, OC43 had a unique role, with seropositive participants of all ages having higher OC43-Spike specific antibodies than seronegative participants, consistent with previous observations [20,33].

A notable finding was that anti-S2 antibodies of SARS-CoV-2, in contrary to antibodies targeting other antigens of SARS-CoV-2, were elevated in seronegative children compared with adolescents and adults. Elevated levels of anti-S2 antibodies, which are more homologous to subunit S2 of seasonal HCoV than subunit S1, may be due to more recent and more frequent exposure to seasonal HCoV. Others have indicated the elevated levels of anti-S2 antibodies to be a possible explanation for the reduced severity of SARS-CoV-2 infections in children [24,33,34].

A key strength of our study stems from the large number of samples tested spanning wide age ranges, and the implementation of different assays including a pseudo functional assay. This study provides a detailed characterization of antibodies to SARS-CoV-2 in one of the largest collection of samples from children assembled to date [35,36]. There was high agreement between the different immunoassays, with all showing a high sensitivity and specificity [26,27], which was confirmed among 90 pre-pandemic pediatric samples in our study. Despite the high degree of correlation assays, a number of samples classified as seronegative by our bead-based and LuLISA assays exhibited pseudoneutralisation activity. The measured neutralising activity in these samples could stem from other antibody isotypes [37].

Seroprevalence estimates are highly dependent on recruitment strategies and should be interpreted carefully as participants, of whom we had limited data, were recruited through residual serum sampling of non-COVID-19 patients in hospitals of which some were located in heavily affected areas of France [30,31]. We did not have data on the medical status of patients whom provided serum samples and therefore limited insight into potential biases. As we used residual samples from hospitals, our study sample is most likely different in terms of overall health compared with the general population and our seroprevalence estimates should therefore not be seen as representative for the general population in north-eastern France. Regarding cross-reactivity, we only focused on antibody levels to whole Spike of the HCoV, cross-reactivity to other antigens was not assessed. Others have described cross-reactivity to Nucleocapsid [24,33]. Finally, potential protection due to previous HCoV infections might also be mediated through cellular immunity [38], which we did not assess in our study.

There has been much discussion regarding the potential role of cross-protective immunity to SARS-CoV-2 infection due to previous seasonal HCoV infection [20,22,24,30], similar to the recently reported interaction between rhinovirus and influenza virus [39]. A detailed understanding of cross-protection of coronaviruses requires appropriately designed studies, such as longitudinal cohorts or experimental infection studies, ideally with assessment of both humoral and cellular immunity. Although large cross-sectional studies such as ours are not appropriately designed to definitively answer questions related to cross-protection, they generate evidence that is consistent with certain hypotheses. Notably, we find evidence for significant cross-reactivity between antibodies to SARS-CoV-2 and seasonal HCoV, but no significant evidence for cross-protective immunity to SARS-CoV-2 infection due to previous seasonal HCoV infection. In particular, across all age groups we did not observe SARS-CoV-2 infected individuals with low levels of antibodies to seasonal HCoV.

## Contributors

MW, AF, SvdW, CD, BH, PC developed the study concept. TW, SP, FA, MA, FD, MW contributed to data curation. AG, CL, HS, RG, CD, KS, JM, EB, SC, OA, JS, FD, DM, CGG, MCB, BMIM, ACD, CS, AG, SL, AJ, HH, AC, FB, MFCV, MSA, and LA collected data. SP, FA, MA, FD performed serological experiments. TW, SP, MW analysed the data and TW created visualizations. LA and PC managed and coordinated research activities. BH, CD, SvdW, AF, and MW acquired financial support. PC, BH, CD, SvdW, AF, and MW supervised the project. SP, FA, MA, FD, and CD validated data and experiments. TW and MW drafted the initial draft. TW, SP, and MW verified the underlying data. All authors reviewed and approved of the final manuscript.

## Data sharing statement

All data and code are available at https://github.com/tomwoudenberg/seroPED. Please inform the authors when using data and code.

## Declaration of Competing Interest

MTW and SPel are inventors on provisional patent PCT/US 63/057.471 on a serological antibody-based diagnostics of SARS-CoV-2 infection. Dr. Dubos reports grants from Universite de Lille, during the conduct of the study. Dr. van der WERF reports grants from Agence Nationale de la Recherche, grants from European Union's Horizon 2020 research and innovation programme, during the conduct of the study; In addition, Dr. van der WERF has a patent USE OF PROTEINS AND PEPTIDES CODED BY THE GENOME OF A NOVEL STRAIN OF SARS ASSOCIATED CORONAVIRUS issued, and a patent SEVERE ACUTE RESPIRATORY SYNDROME (SARS) - ASSOCIATED CORONAVIRUS DIAGNOSTICS pending.

## Appendix Supplementary materials

Image, application 1
