# Supplementary material for: Humoral immunity to SARS-CoV-2 and seasonal coronaviruses in children and adults in north-eastern France
Source: eBioMedicine. 2021 Jul 23;70:103495. doi: 10.1016/j.ebiom.2021.103495 (PMC8299153; doi:10.1016/j.ebiom.2021.103495)
Supplement: Supplementary file 1 [file mmc1.docx]

**Supplementary Table and Figures**


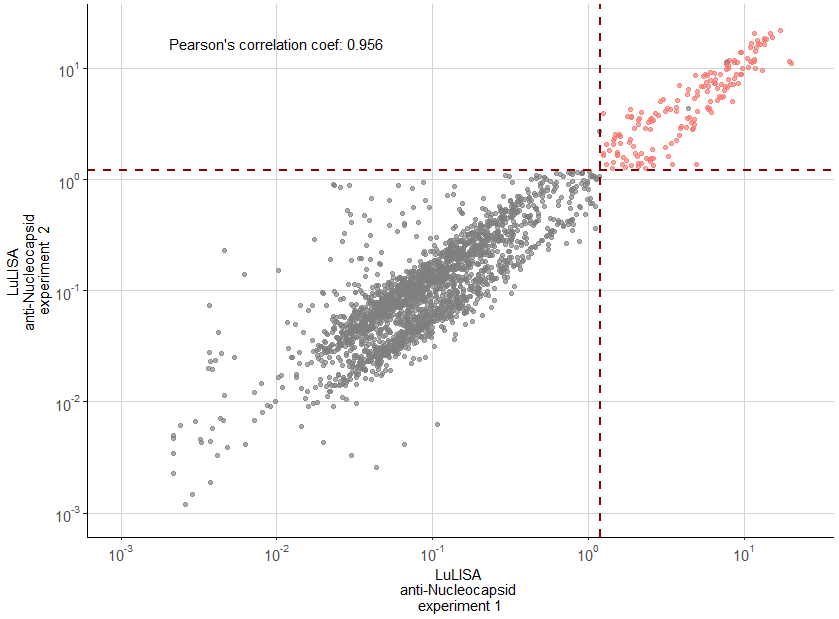


Supplementary Figure 1. Antibody responses among samples from adults and children hospitalized for other reasons than COVID-19. Antibodies are Nucleocapsid-specific and measured twice with the Luciferase-Linked ImmunoSorbent Assay.

| Table 1. Concordance assessment between the bead-based multiplex assay (Luminex), Luciferase-Linked ImmunoSorbent Assay (LuLISA), and pseudoneutralising assay | | | | | |
| --- | --- | --- | --- | --- | --- |
| Test | Compared to | Overall agreement | Positive percent agreement | Negative percent agreement | Cohen’s Kappa statistic |
| Luminex | LuLISA | 95.3;  2296 / 2408 | 65.7;  115 / 175 | 97.7;  2181 / 2233 | 0.65 (0.59 - 71) |
|  | Pseudo-neutralisation | 95.2;  2277/2392 | 73.4;  127 / 173 | 96.9;  2150 / 2219 | 0.66 (0.60 – 0.72) |
| LuLISA | Luminex | 95.3;  2296 / 2408 | 68.9;  115 / 167 | 97.3;  2181 / 2241 | 0.65 (0.59 - 71) |
|  | Pseudo-neutralisation | 94.6;  2303 / 2434 | 70.4;  114 / 162 | 96.3  2189 / 2272 | 0.61 (0.54 -0.67) |
| Pseudo-neutralisation | Luminex | 95.2;  2277 / 2392 | 64.8;  127 / 196 | 97.9;  2150 / 2196 | 0.66 (0.60 – 0.72) |
|  | LuLISA | 94.6;  2303 / 2434 | 57.9;  114 / 197 | 97.9;  2189/2237 | 0.61 (0.54 -0.67) |


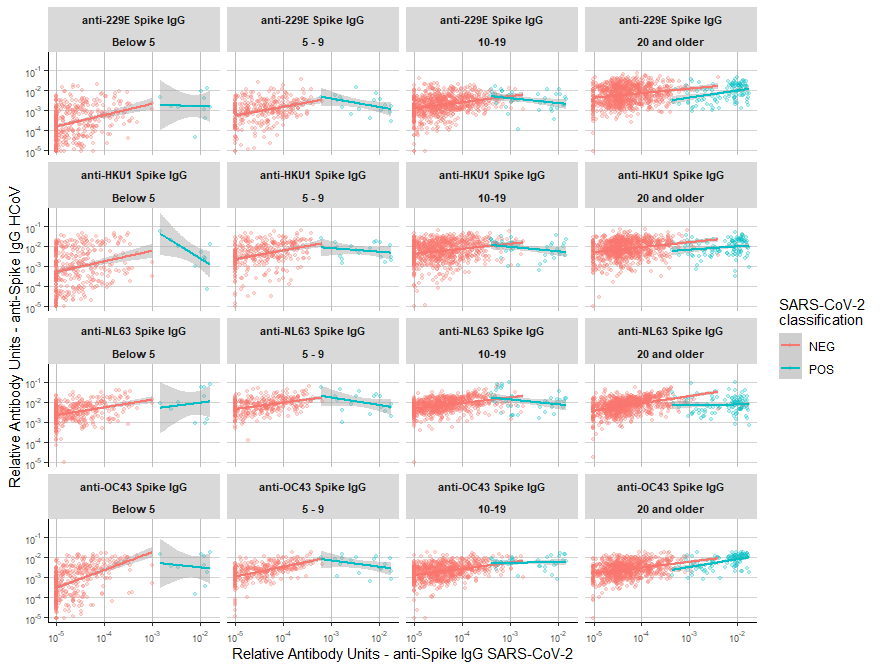


Supplementary Figure 2. Correlation between Spike IgG antibodies to SARS-CoV-2 and Spike IgG antibodies to four human coronaviruses (HCoV) stratified by age and SARS-CoV-2 seropositivity.

**Appendix**

*Development algorithm*

In order to classify the samples from the participants as seropositive or seronegative with our 9-plex bead-based immunoassay, we developed an algorithm using negative controls consisting of pre-epidemic samples (n = 407) and positive controls, PCR-confirmed infections (n = 970) (1, 2).

Distribution of responses (Figure 3a-c) and pairwise comparisons (Figure 3e-g) of antibodies to different antigens are visualized in Figure 3. Antibodies-targeting SARS-CoV-2 Spike yielded the highest area under the curve followed by Recepter Binding Domain and Nucleocapsid-specific antibodies (Figure 3d).

The developed algorithm was a random Forest using 3 different antigens. The performance of this algorithm was re-adjusted to a specificity of 99%. With this specificity target, the test yielded a sensitivity of 98%. Uncertainty was assessed by cross validating this performance, resulting in a sensitivity ranging from 93 to 100%.


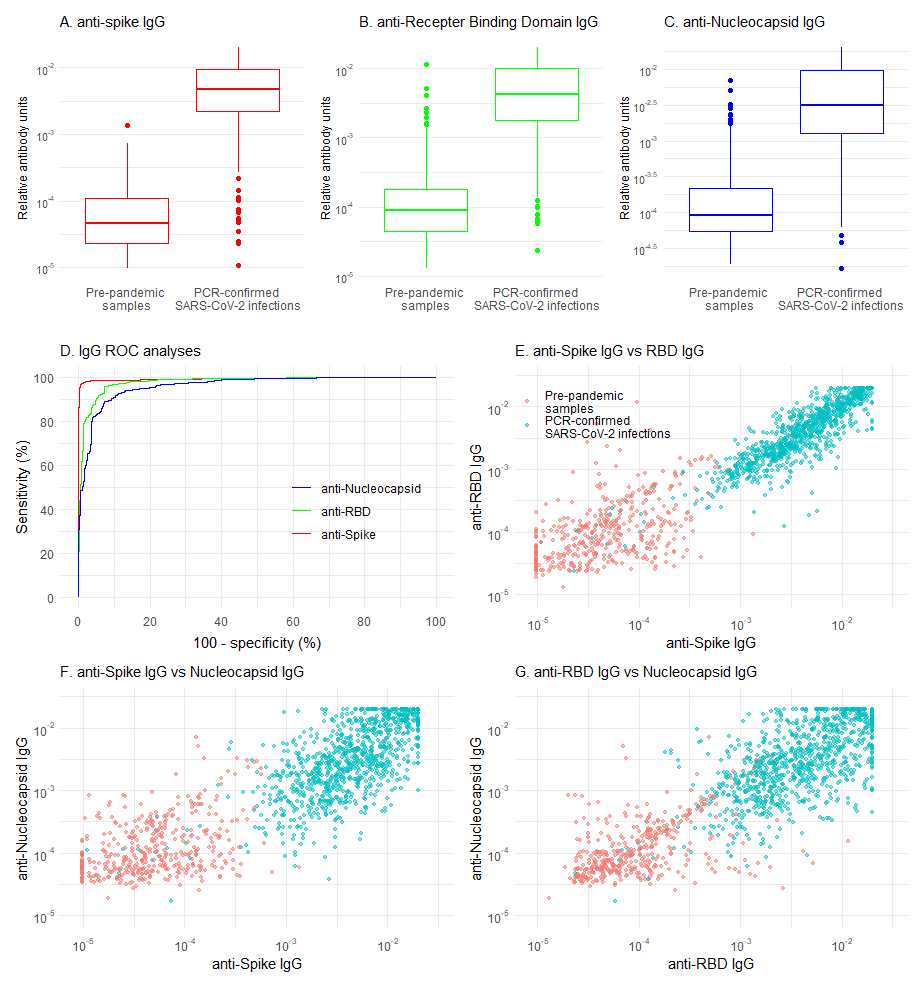


Supplementary Figure 3. Overview of antibody responses of samples from positive and negative panels and classification performance. Panel a-c show the antibody distribution measured stratified by SARS-CoV-2 status for anti-Spike, anti-RBD, and anti-NP IgG. Panel e-g show the pairwise combinations of antibody responses. Panel d shows the ROC-curve, which evaluates the classification performance of three single antigens: anti-Spike, anti-Nucleocapsid, and anti-RBD IgG.

1. Rosado, J., Pelleau, S., Cockram, C., Merkling, S. H., Nekkab, N., Demeret, C., ... & White, M. T. (2021). Multiplex assays for the identification of serological signatures of SARS-CoV-2 infection: an antibody-based diagnostic and machine learning study. *The Lancet Microbe*, *2*(2), e60-e69.
2. Pelleau, S., Woudenberg, T., Rosado, J., Donnadieu, F., Garcia, L., Obadia, T., ... & White, M. (2021). Serological reconstruction of COVID-19 epidemics through analysis of antibody kinetics to SARS-CoV-2 proteins. *medRxiv*.

*Overview reagents, cell lines and antibodies.*

| Reagent / cell line, model organism / antibodies | Source (including supplier name) | Catalog number / accession number | RRID if available |
| --- | --- | --- | --- |
| **bead-based 9-plex assay (Luminex)** | | |  |
| R-Phycoerythrin-(R-PE) conjugated Donkey Anti-Human IgG | Jackson Immunoresearch | 709-116-098 | AB_2340519 |
| SARS-CoV-2 trimeric Spike ectodomain | Structural virology unit, Institut Pasteur |  |  |
| SARS-CoV-2 Receptor Binding Domain | Structural virology unit, Institut Pasteur |  |  |
| SARS-CoV-2 Spike S2 subunit | Native antigen | REC31807-100 |  |
| SARS-CoV-2 Nucleocapsid | Recombinant protein platform, Institut Pasteur |  |  |
| SARS-CoV-2 Membrane–Envelope fusion | Native antigen | REC31848-100 |  |
| 229E trimeric Spike | Structural virology unit, Institut Pasteur |  |  |
| HKU1 trimeric Spike | Structural virology unit, Institut Pasteur |  |  |
| NL63 trimeric Spike | Structural virology unit, Institut Pasteur |  |  |
| OC43 Spike | Sino Biological | 40607-V08B-100 |  |
| **PNT assay** | | | |
| DMEM High Glucose, HEPES, No Phenol, L-Glutamine 500ml | GIBCO | 21063-029 |  |
| Penicilin/Streptomycin 100X | GIBCO | 15140-122 |  |
| FBS | CORNING | 35-079-CV |  |
| DPBS | GIBCO | 14199-094 |  |
| EDTA | Promega | H5032 |  |
| ONE-Glo™ Luciferase Assay System | Promega | E6110 |  |
| 96-well sterile cell culture plate with flat bottom, Black | Greiner | 655090 |  |
| HEK 293T hACE2 | Institut Pasteur -Theravectys |  |  |
| Spike-pseudotyped Lentiviral Vector | Institut Pasteur -Theravectys |  |  |
| **Luciferase-Linked ImmunoSorbent assay (LuLISA)** | | | |
| Full-length N protein | Stéphane Petres (Institut Pasteur) |  |  |
| Anti‐Fc IgG VHH (Fc1) | Thierry Rose (Pasteur Institut) |  |  |
| Fc1‐nanoKAZ | Yves Janin (Pasteur Institut) |  |  |

*Consulted articles to write the research in context:*

(“SARS-CoV-2” OR “COVID-19”) AND (“antibody” OR “humoral immunity”) AND (“seasonal coronavirus” OR “OC43” OR “NL63” OR “HKU1” OR “229E”)

| **author** | **Method** | **Result** |
| --- | --- | --- |
| Peterhoff et al. | We assessed sera from patients infected with seasonal coronaviruses, SARS-CoV-2 and controls. | The RBD-based ELISA detected SARS-CoV-2-directed antibodies, did not cross-react with seasonal coronavirus antibodies and correlated with virus neutralization (R2 = 0.89 |
| Iyer | We measured plasma and/or serum antibody responses to the receptor-binding domain (RBD) of the spike (S) protein of SARS-CoV-2 in 343 North American patients infected with SARS-CoV-2 (of which 93% required hospitalization) up to 122 days after symptom onset and compared them to responses in 1548 individuals whose blood samples were obtained prior to the pandemic | We observed no cross-reactivity of the SARS-CoV-2 RBD-targeted antibodies with other widely circulating coronaviruses (HKU1, 229 E, OC43, NL63) |
| **Phuong Nguyen-Contant** | **We analysed sera and peripheral blood mononuclear cells (PBMCs) from non-SARS-CoV-2-exposed healthy donors and COVID-19 convalescent subjects. Serum IgG levels specific for SARS-CoV-2 proteins (S, including the RBD and S2 subunit, and nucleocapsid [N]) and non-SARS-CoV-2 proteins were related to measurements of circulating IgG MBC levels.** | Our findings indicate cross-reactive B cell responses against the S2 subunit that might enhance broad coronavirus protection |
| **Loos** | We profiled the immune responseacross multiple coronavirus receptor binding domains (RBDs), respiratory viruses, andSARS-CoV-2, to determine whether heterologous immunity to other CoV-RBDs orother infections influenced the evolution of the SARS-CoV-2 humoral immune re-sponse | Moreover, therewas little evidence of correlation between SARS-CoV-2 responses and HKU1, NL63,and respiratory infection (influenza and respiratory syncytial virus) responses. These findings suggest that common viral infections including common CoV immunity, tar-geting the receptor binding domain involved in viral infection, do not appear to in-fluence the rapid functional evolution of SARS-CoV-2 immunity |
| **Hicks** | **Here, we discuss the cross-reactivity potential of SARS-CoV-2 antibodies with the full spike proteins of four other betacoronaviruses thatcause disease in humans, MERS-CoV, SARS-CoV, HCoV-OC43, and HCoV-HKU1.** |  |
| **Charlton** | High- to mid-volume enzyme immunoassays were also evaluated with a separate panelof convalescent-phase sera to evaluate cross-reactivity to common respiratory virusesand non-SARS-CoV-2 coronaviruses | **Max 2 (**CoV-229E[n2], CoV-NL63 [n2]**) out of 65 on one of the six evaluated EIAs.** |
| **Secchi** | 509 patients confirmed to have COVID-19 from the San Raffaele Hospital of Milan and 480 samples of prepandemic organ donor sera collected in 2010–2012. Using fluid-phase luciferase immune precipitation (LIPS) assays, we characterized IgG, IgM, and IgA antibodies to the spike receptor binding domain (RBD), S1+S2, nucleocapsid, and ORF6 to ORF10 of SARS-CoV-2, to the HCoV-OC43 and HCoV-HKU1 betacoronaviruses spike S2, and the H1N1Ca2009 flu virus hemagglutinin. | We observed a major synchronous expansion of antibodies to the HCoV-OC43 and HCoV-HKU1 spike S |
| **Ladner** | we study cross-reactivity using a highly multiplexed peptide assay (PepSeq) to generate an epitope-resolved view of IgG reactivity across all human CoVs in both COVID-19 convalescent and negative donors. (n = 110, adults) | we demonstrate that SARS-CoV-2 elicits antibodies that cross-recognize pandemic and endemic CoV antigens at two Spike S2 subunit epitopes. |
| **Song** | We compared serum antibody and memory B cell responses to coronavirus spike (S) proteins from pre-pandemic and SARS-CoV-2 convalescent donors using a series of binding and functional assays. (n = 36 controls and convalescent sera) | We found weak evidence of pre-existing SARS-CoV-2 cross-reactive serum antibodies in pre-pandemic donors. However, we found stronger evidence of pre-existing cross-reactive memory B cells that were activated on SARS-CoV-2 infection. Monoclonal antibodies (mAbs) isolated from the donors showed varying degrees of cross-reactivity with betacoronaviruses, including SARS and endemic coronaviruses. None of the cross-reactive mAbs were neutralizing except for one that targeted the S2 subunit of the S protein. |
| **Liu** | Here, we report antibody and antibody-avidity assays, to detect antibodies to the S1 subunit of the spike protein and to the RBD of SARS-CoV-2 in human serum and saliva, and for quantifying immunoglobulin avidities against coronavirus antigens from SARS-CoV-2, SARS-CoV-1 and the common-cold viruses OC43, HKU1, NL63 and 229E. | In pre-pandemic serum samples, antibodies against the com-mon colds did not cross-react with SARS-CoV-2 or SARS, except for one sample showing detectable IgG against SARS S1 |
| **Simula** | ? | ? |
| **Henss** | In the current study, humoral immune responses were characterized in a cohort of 143 patients. SARS-CoV-2-specific-antibodies were detected by enzyme-linked immunosorbent assay (ELISA). SARS-CoV-2 and human coronavirus NL63 neutralization activity was analysed with pseudotyped lentiviral vectors. | high NL63 neutralizing activity was not detected in samples from patients with severe COVID-19 (score of 5-7) patients |
| **Laing** | we developed and applied a multiplex microsphere-based immunoassay(MMIA) for COVD-19 antibody studies that incorporates spike protein trimers of SARS-CoV-2, SARS-CoV-1,MERS-CoV, and the seasonal human betacoronaviruses, HCoV-HKU1 and HCoV-OC43, that enables measurement of off-target pre-existing cross-reactive antibodies. | HCoV-OC43 IgG antibody levels was detected with SARS-CoV-2 seroconversion in a subset of subjects for whom early infection sera were available prior to their SARS-CoV-2seroconversion, suggestive of an HCoV-OC43 memory response triggered by SARS-CoV-2 infection |
| **Karp** | Here, we present an ultrasensitive and high-throughput automated liquid biopsy assay based on the Hamilton Microlab ADAP STAR automated liquid-handling platform, which was developed and validated for the qualitative detection of total antibodies against spike protein 1 (S1) of SARS-CoV-2 that uses as little as 4 µL of serum. To assess the clinical performance of the ADAP assay, 57 PCR-confirmed COVID-19 patients and 223 control patients were tested | Notably, the SARS-CoV-2–negative control patients included individuals with other common coronaviral infections, such as CoV-NL63 and CoV-HKU, which did not cross-react. |
| **Alandijany** | SARS-CoV-2 full-length spike (S) recombinant protein was utilized to develop and optimize an indirect enzyme-linked immunoassay (ELISA) that enables a reliable detection of virus-specific IgG antibody in human sera. | The assay specifically detects human IgG antibodies directed against SARS-CoV-2, but not those to Middle East respiratory syndrome coronavirus (MERS-CoV) or human coronavirus HKU1 (HCoV-HKU1). |
| **Ruetalo** | We conducted a comprehensive serological survey of 49 patients with a mild course of disease and quantified neutralizing antibody responses against a clinical SARS-CoV-2 isolate employing human cells as targets. | In addition, employing a novel serological Western blot system to characterize antibody responses against seasonal coronaviruses, we found that antibodies against the seasonal coronavirus 229E might contribute to SARS-CoV-2 neutralization. |
| **Guo** | In this study, we profiled the temporal changes of IgG antibody against spike proteins (S-IgG) of SARS-CoV-2 and seasonal HCoVs in 838 plasma samples collected from 344 COVID-19 patients. | We found that SARS-CoV-2 S-IgG titres mounted until days 22-28, whereas HCoV-OC43 antibody titres increased until days 15-21 and then plateaued until day 46. However, IgG titres against HCoV-NL63, -229E, and -HKU1 showed no significant increase. A two-way cross-reactivity was identified between SARS-CoV-2 and HCoV-OC43. Neutralizing antibodies against SARS-CoV-2 were not detectable in healthy controls who were positive for HCoV-OC43 S-IgG. |
| **Gaudelus** | We tested if prior infections by seasonal coronaviruses (HCoV) NL63, HKU1, 229E or OC43 as assessed by serology, provide cross-protective immunity against SARS-CoV-2 infection | Prior infection by seasonal coronaviruses, as assessed by serology, does not interfere with SARS-CoV-2 infection and related MIS in children |
| **Petrie** | We investigated frequency of reinfection with seasonal coronaviruses (HCoV) and serum antibody response following infection over 8 years in the Household Influenza Vaccine Evaluation cohor | Antibody levels were also measured against SARS-CoV-1 spike protein, SARS-CoV-2 spike protein, SARS-CoV-2 spike protein receptor binding domain, SARS-CoV-2 spike protein N-terminal domain, and SARS-CoV-2 N protein (Table 3). With the exception of the SARS-CoV-2 N protein, antibody levels were low against these targets and did not substantially change from pre-to post-infection with each of the 4 seasonal coronaviruses. |
